# Activation of the native *PHYTOENE SYNTHASE 1* promoter by modifying near-miss *cis*-acting elements induces carotenoid biosynthesis in embryogenic rice callus

**DOI:** 10.1007/s00299-024-03199-7

**Published:** 2024-04-17

**Authors:** Guillermo Sobrino-Mengual, Derry Alvarez, Richard M. Twyman, Christopher Gerrish, Paul D. Fraser, Teresa Capell, Paul Christou

**Affiliations:** 1 Applied Plant Biotechnology Group, Department of Agricultural and Forest Sciences and Engineering, University of Lleida-Agrotecnio CERCA Center, Lleida, Spain; 2 TRM Ltd, PO Box 493, Scarborough, YO11 9FJ UK; 3 ICREA, Catalan Institute for Research and Advanced Studies, Barcelona, Spain; 4 Department of Biological Sciences, Royal Holloway University of London, Egham Hill, Egham, Surrey TW20 0EX UK; 5 Present Address: Division of Biological and Environmental Sciences and Engineering, Center for Desert Agriculture, BioActives Lab, King Abdullah University of Science and Technology (KAUST), Thuwal, Saudi Arabia

**Keywords:** Callus, Carotenoids, *cis*-acting element, Endosperm, Metabolic engineering, Promoter

## Abstract

**Key message:**

**Modification of silent latent endosperm-enabled promoters (SLEEPERs) allows the ectopic activation of non-expressed metabolic genes in rice callus**

**Abstract:**

Metabolic engineering in plants typically involves transgene expression or the mutation of endogenous genes. An alternative is promoter modification, where small changes in the promoter sequence allow genes to be switched on or off in particular tissues. To activate silent genes in rice endosperm, we screened native promoters for near-miss *cis*-acting elements that can be converted to endosperm-active regulatory motifs. We chose rice *PHYTOENE SYNTHASE 1* (*PSY1*), encoding the enzyme responsible for the first committed step in the carotenoid biosynthesis pathway, because it is not expressed in rice endosperm. We identified six motifs within a 120-bp region, upstream of the transcriptional start site, which differed from endosperm-active elements by up to four nucleotides. We mutated four motifs to match functional elements in the endosperm-active *BCH2* promoter, and this promoter was able to drive *GFP* expression in callus and in seeds of regenerated plants. The 4 M promoter was not sufficient to drive *PSY1* expression, so we mutated the remaining two elements and used the resulting 6 M promoter to drive *PSY1* expression in combination with a *PDS* transgene. This resulted in deep orange callus tissue indicating the accumulation of carotenoids, which was subsequently confirmed by targeted metabolomics analysis. *PSY1* expression driven by the uncorrected or 4 M variants of the promoter plus a *PDS* transgene produced callus that lacked carotenoids. These results confirm that the adjustment of promoter elements can facilitate the ectopic activation of endogenous plant promoters in rice callus and endosperm and most likely in other tissues and plant species.

**Supplementary Information:**

The online version contains supplementary material available at 10.1007/s00299-024-03199-7.

## Introduction

Metabolic engineering in plants involves the modulation of biosynthetic pathways to increase or decrease the abundance of particular molecules or to produce new ones (DellaPenna 2001; Zhu et al. 2007). This usually requires the expression and/or suppression of specific enzymes. Loss-of-function approaches, such as conventional or insertional mutagenesis, RNA interference or gene editing, aim to abolish enzyme functions, restricting or blocking particular pathways and thus causing the accumulation or diversion of intermediates. In contrast, gain-of-function approaches usually involve the overexpression of transgenes encoding rate-limiting enzymes, either native sequences or engineered versions with modified activities (Zhu et al. 2008; Bai et al. 2016; Zhai et al. 2016). Such transgenes are typically driven by strong constitutive, tissue-specific or inducible promoters (Peremarti et al. 2010).

In cereal plants, metabolic engineering has been used to increase the levels of nutritionally relevant carotenoids in the endosperm. Examples include Golden Rice, which accumulates β-carotene (provitamin A) (Paine et al. 2005), and Carolight maize, which produces β-carotene (Naqvi et al. 2009) and a broad spectrum of additional carotenoids (Zhu et al. 2018). The main target in both products was the enzyme *PHYTOENE SYNTHASE *1 (PSY1), which is responsible for the first committed step in the carotenoid biosynthetic pathway. The *PSY1* gene is not expressed in rice endosperm or in white-kernel maize varieties such as M37W, from which Carolight maize was derived. Golden Rice and Carolight maize addressed this issue by expressing a *PSY1* transgene together with bacterial phytoene desaturase (*CRTI*) to boost the early part of the pathway, each under the control of a strong endosperm-specific promoter, thus effectively removing the bottleneck in the endosperm carotenoid pathway.

An alternative to transgene cassettes for the expression of inactive endogenous genes is in situ promoter modification. Whereas transgenes carry their own promoters, in situ modification introduces small changes in the upstream promoter region of native genes to modulate promoter activity. Such modifications are indistinguishable from natural mutations and indeed recapitulate a major evolutionary mechanism for the adaptation of plants in which regulatory mutations influencing gene expression levels lead to subtle changes in phenotype rather than dramatic changes caused by structural mutations (Vedel and Scotti 2011). The first report of in situ promoter modification in plants was the creation of allelic series in the *CLAVATA3* gene controlling fruit size in tomato (Rodriguez-Leal et al. 2017). However, that study used genome editing to vary the expression level of an active gene, whereas *PSY1* is inactive in rice endosperm and there have been no reports thus far in which in situ promoter modification has been used for the ectopic activation of silent genes. Our long-term approach is to modify native promoters that are inactive in the endosperm by using genome editing to convert near-miss *cis*-acting elements into functional counterparts, thus awakening “silent latent endosperm-enabled promoters” (SLEEPERs).

The success of the SLEEPER approach requires an understanding of the structure of endosperm-specific promoters in cereals (Kawakatsu et al. 2008). Several *cis*-acting elements are commonly found in cereal endosperm-specific promoters (Onodera et al. 2001), including the prolamin box (P-box)/GCN4 core motif, AACA motif, and Opaque 2 (O2) motif (Chuan-Yin et al. 1998; Wu et al. 2000; Kawakatsu et al. 2008; Jin et al. 2019). These are bound by transcription factors of the DOF zinc finger, MYB and bZIP families, respectively (Juhász et al. 2011). We previously found that the P-box and AACA motifs in the promoter of the maize *BCH2* gene (encoding another enzyme in the carotenoid pathway) could activate reporter genes independently and additively in maize and rice endosperm, but the transcription factors that bind these elements were unable to boost expression of the maize *PSY1* gene, which is active in the endosperm of yellow maize (Jin et al. 2019).

We selected the inactive rice *PSY1* promoter as our first target because callus expressing *PSY1* and *PDS* accumulates carotenoids, resulting in an easily scored colored callus phenotype. Furthermore, the method can be verified without the need to regenerate transgenic plants because embryogenic callus is permissive for the expression of endosperm-specific genes (Bai et al. 2014). We screened for motifs within the *PSY1* promoter differing by up to four nucleotides from endosperm-specific *cis-*acting elements, and six candidates were found within a 120-bp region ~ 300 bp upstream of the transcriptional start site. We then prepared constructs in which four of these sequences (matching those in the maize *BCH2* promoter) were corrected and attached to the *GFP* reporter gene for proof-of principle testing in transgenic callus, and constructs with four or six corrected sequences were then used to drive the *PSY1* gene for metabolic engineering. This cautious approach was necessary to check that the juxtaposition of the corrected elements satisfies the structural requirements of an endosperm-enabled promoter before progressing to the in situ modification of promoters by genome editing.

## Results

### Analysis of *cis*-regulatory elements in the rice *PSY1* promoter in silico

**Fig. 1:**
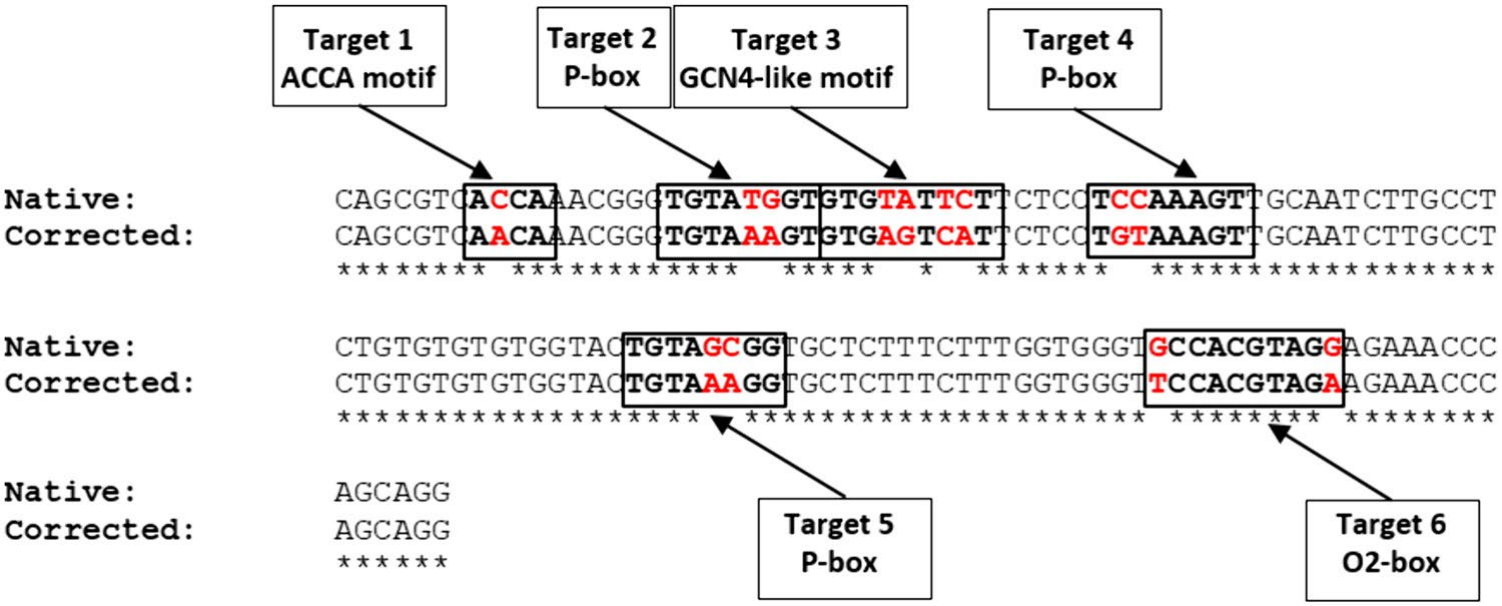
Schematic representation of the native and corrected rice *PSY1* promoter region. The native and corrected sequences are shown in a ~ 120-bp segment of the *PSY1* promoter (−300 region) that contains six target sites (red boxes) resembling *cis*-acting regulatory elements that confer endosperm-specific expression. Each site can be corrected (changed to an active sequence) by mutating 1–4 bases (highlighted in red)

The rice *PSY1* gene is not expressed in the endosperm because the native promoter lacks the appropriate *cis*-acting regulatory elements (Fauteux and Strömvik 2009). However, other genes encoding downstream enzymes in the same pathway *are* expressed in the endosperm, and it is possible that ancestors of modern rice had a functional carotenoid pathway in the endosperm but have lost this capability due to the inactivation of the early steps (Chettry and Chrungoo 2020). The expression of native rice *PSY1* under the control of a heterologous promoter can restore the pathway when combined with PDS, indicating that the deficiency is located in the *PSY1* regulatory elements not the coding region. We therefore hypothesized that the native *PSY1* promoter may contain partial matches to binding sites for endosperm-specific transcription factors, perhaps even remnants of ancient motifs reflecting the evolutionary loss of promoter activity, and that these could converted into functional elements leading to tissue-specific activation. We used a BLAST search to screen the 2.5-kb upstream promoter region of the rice *PSY1* gene for such partial matches, and identified six candidate sites within a 120-bp region ~ 300 bp upstream of the transcriptional start site (Fig. 1). These comprised three P-boxes, one AACA motif, one GCN4-like motif and an O2 box, each with 1–4 mismatched bases (Table 1).

**Table 1:** Summary of the target motifs

Target site	Motif	Native sequence (5′ → 3′)	Corrected sequence (5′ → 3′)
1 (GFP)	AACA motif	ACCAAAC	A*A*CAAAC
2 (GFP)	(P-box) (-300 ELEMENT)	TGTATGGT	TGT*AA*AGT
3	GCN4-like motif (GZM)	GTGTATTCT	GTG*AG*T*CA*T
4 (GFP)	(P-box) (-300 ELEMENT)	TCCAAAGT	T*GT*AAAGT
5 (GFP)	(P-box) (-300 ELEMENT)	TGTAGCGG	TGTA*AA*GG
6	(O2-box)	GCCACGTAGG	*T*CCACGTAG*A*

Italic letters indicate the bases to be corrected in each motif

### Design of corrected *PSY1* promoters and preparation of expression constructs

We cloned 2.5 kb of the 5′-flanking region from the rice *PSY1* gene (GenBank FJ971175.1) from leaf genomic DNA (*Oryza sativa* cv. Nipponbare) (Supplementary Fig. 1). Four of the near-miss elements resembled three P-boxes and one AACA motif, a configuration which we previously found sufficient to activate the maize *BCH2* gene (but not the maize or rice *PSY1* genes) by expressing the corresponding transcription factors (Jin et al. 2019). New sequences were, therefore, generated in which four or all six of the native motifs were corrected to form active transcription factor binding sites (Table 1). The native and corrected promoters were compared using PLACE software (Higo et al. 1999) to predict the outcome of the modifications (Supplementary Fig. 2). The promoter with four corrected sequences (4 M) was attached to the *GFP* reporter gene for proof-of-principle testing and subsequently to the *PSY1* gene to investigate its effect on carotenoid metabolism. The other two motifs were also corrected to generate the 6 M promoter, which was attached to the *PSY1* gene to determine whether the additional elements would boost its expression.

### Transformation of rice with native and corrected *PSY1* promoters controlling *GFP*

To test the concept of SLEEPER reactivation, we prepared constructs in which the *gfp* marker gene (encoding plant-optimized green fluorescent protein) was controlled by either the “uncorrected” native *PSY1* promoter (Na) or the 4 M promoter in which four near-miss *cis*-acting elements were converted into three active P-boxes and one AACA motif similar to the maize *BCH2* promoter (Table 1). A further construct was prepared in which the selectable marker *hpt* was controlled by the constitutive maize ubiquitin-1 promoter and first intron (*Ubi-1*). All constructs also featured the Nos terminator (TNos). The constructs 4 M-*GFP*-TNos and Na-*GFP*-TNos were introduced separately, each paired with the *Ubi-1-hpt* construct. Callus lines were analyzed by PCR using primers overlapping the promoter region and *GFP* transgene to identify transformants.

### Expression profiles of *GFP* mRNA and GFP protein

**Fig. 2:**
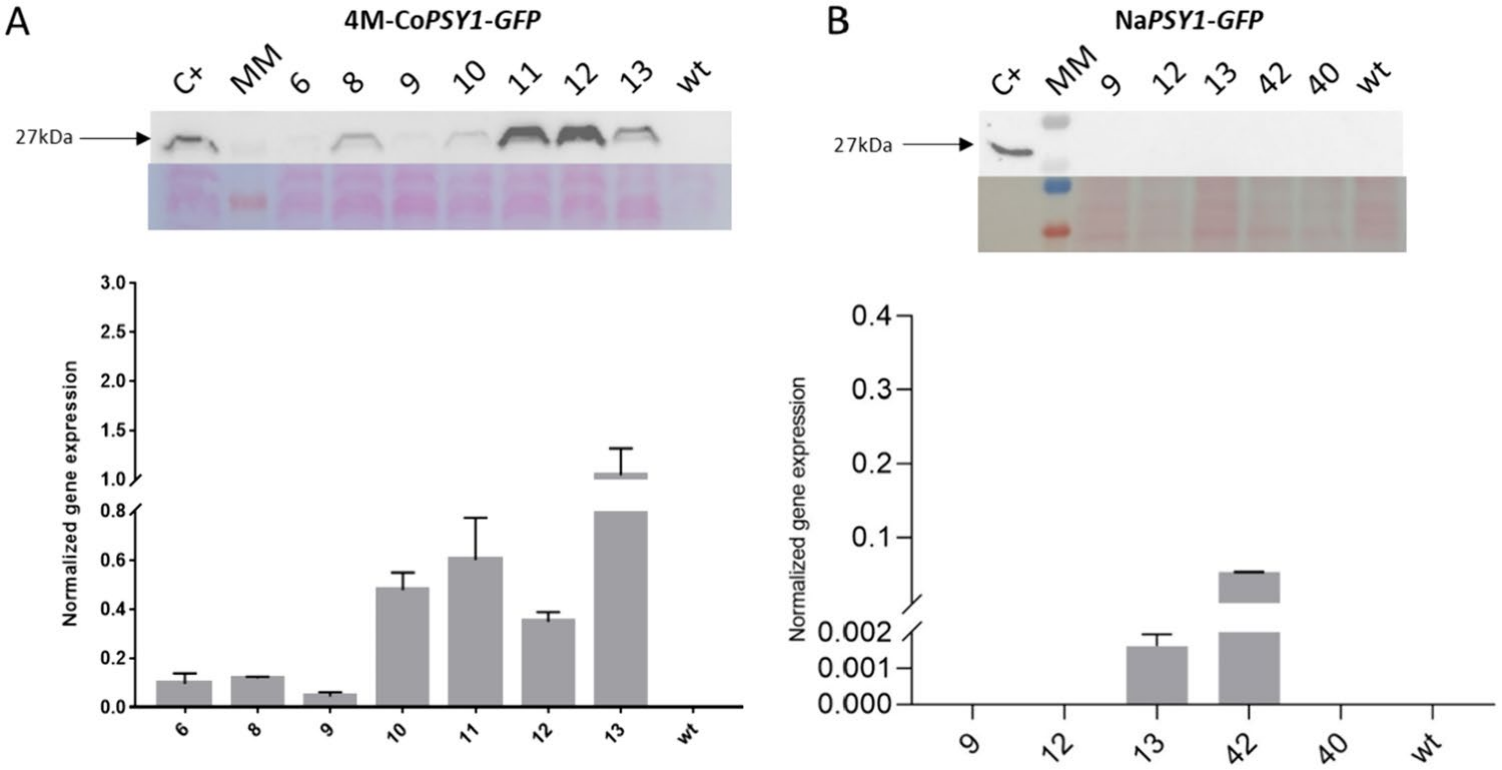
*GFP* transgene expression and western blot analysis. **A** Lanes 6, 8, 9, 10, 11, 12 and 13 represent independent 4 M-*GFP*-TNos lines. **B** Lanes 9, 12, 13, 42 and 40 represent independent Na-*GFP*-TNos lines (wt = wild-type rice callus as a negative control). The expression of *GFP* was measured by qRT-PCR in rice callus. Expression levels were normalized against the rice *actin* gene. Each value represents the mean of three different biological replicates and error bars represent standard deviations

**Fig. 3:**
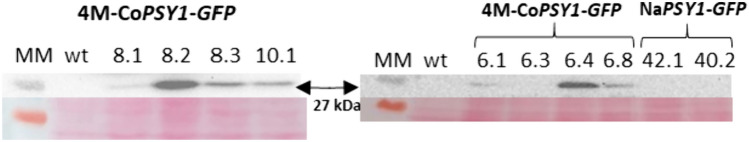
Western blot analysis of T1 rice seeds to detect GFP. **A** Lanes 8.1, 8.2, 8.3 and 10.1 and (**B**) 6.1, 6.3, 6.4 and 6.8 represent independent lines of 4 M*-GFP*-TNos. **B** Lanes 42.1 and 40.2 represent independent lines of Na-*GFP*-TNos (wt = wild-type rice callus as a negative control)

The expression profiles of constructs 4 M-*GFP*-TNos and Na-*GFP*-TNos in transgenic callus lines were analyzed by quantitative real-time PCR (qRT-PCR; Fig. 2A, B). The 4 M promoter increased the relative expression of *GFP* from basal levels (0.1–0.2) to 0.8–1 in some of the lines (Fig. 2A, B). GFP protein was detected using an anti-GFP antibody, revealing protein bands with the expected molecular mass of 27 kDa (Fig. 2A, B). T1 seeds from independent transgenic lines regenerated from the callus were analyzed to determine whether GFP was present (Fig. 3). Most lines transformed with the 4 M promoter construct accumulated GFP (Fig. 3A, B) whereas lines transformed with the native *PSY1* promoter did not (Fig. 3B).

### Confirmation of GFP activity in T1 seeds by confocal microscopy

**Fig. 4:**
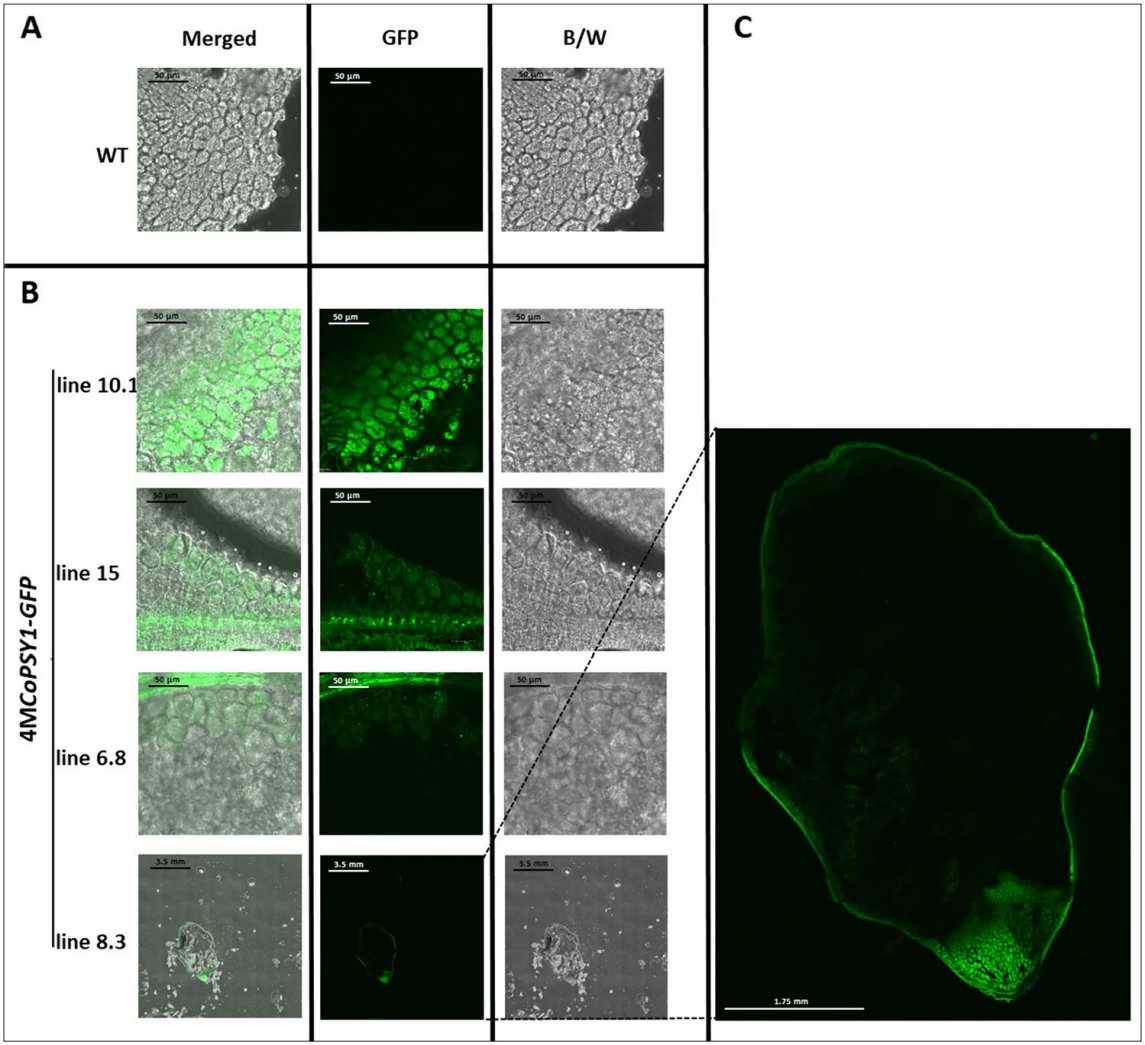
Confocal images of seed cross sections from wild-type (WT) plants and 4 M-*GFP* transgenic lines. **A** WT lines. **B** 4 M*-GFP* lines. **C** Detail of the transverse section of 4 M*-GFP* line 8.3. The images of 4 M-*GFP* lines 10.1, 15 and 6.8 have the same magnification as the WT seed and show cells of the embryo scutellum. Green channel (GFP), bright field (B/W) and Merged (overlay of the above)

**Fig. 5:**
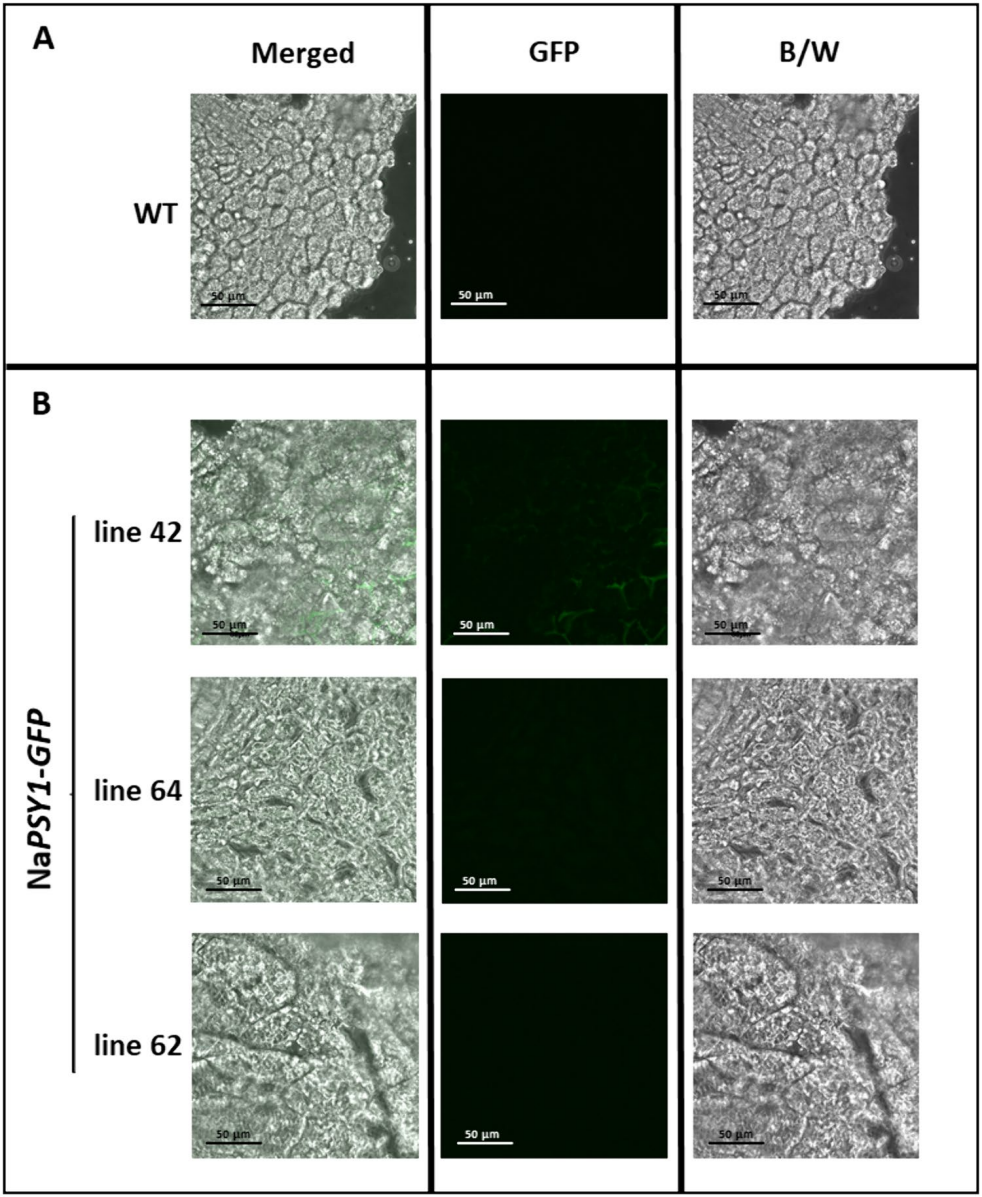
Confocal images of seed cross sections from wild-type (WT) plants and Na-*GFP* transgenic lines. **A** WT lines. **B** Na*-GFP* lines. Green channel (GFP); bright field (B/W) and Merged (overlay of the above two photographs)

The subcellular localization of GFP was evaluated by confocal microscopy in the mature T1 seeds of transgenic lines expressing the 4 M-*GFP*-TNos and Na-*GFP*-TNos constructs, with wild-type (WT) plants as a control. Fluorescence was observed in the cells of the embryo scutellum in all 4 M-*GFP* lines (Fig. 4). At a lower magnification, we observed GFP localization in the seed and seed integument (Fig. 4C, showing line 4 M-*GFP*-8.3 as an example). In contrast, the Na-*GFP* lines showed no evidence of GFP above basal levels in these regions, similar to the WT controls (Fig. 5A, B).

GFP levels determined by western blot (Fig. 3) correlated with the intensity of the cellular GFP signal in the mature seeds of 4 M-*GFP* lines 6.8, 8.3 and 10.1 (Fig. 4). In contrast, most of the Na-*GFP* lines showed no evidence of GFP protein (Fig. 3) or GFP fluorescence in the seeds (Fig. 5A, B). One exception was line Na-*GFP* 42, which despite the absence of GFP in western blots showed background fluorescence in the seeds. Interestingly, this line also accumulated *GFP* mRNA (Fig. 2B) but at very low levels compared with the 4 M-*GFP* lines (Fig. 2A). This is probably a consequence of position effects, reflecting the integration of the Na-*GFP*-TNos cassette at a genomic region that promotes low levels of expression in line 42. Protein levels and subcellular localization data could not be acquired for all lines because not enough seeds were available for both experiments.

### Transformation of rice with native and corrected *PSY1* constructs plus *PDS*

Having established that the 4 M promoter was sufficient to drive GFP expression in the callus and endosperm, we attached it to the *PSY1* gene and evaluated its expression in transgenic callus. Interestingly, we did not see any evidence of *PSY1* expression at the mRNA level. The accumulation of phytoene in the transgenic callus was also negligible, indicating the absence of enzymatic activity. We previously reported that the two transcription factors responsible for trans-activating the P-box and AACA motif in maize were sufficient to induce the expression of *BCH2* but not *PSY1*, suggesting that other factors are also required for significant levels of *PSY1* expression in maize. Given the important role of GCN4 core motifs and O2 motifs in other endosperm-specific promoters, we therefore prepared the 6 M promoter with all six motifs converted to functional binding sites for further testing. Rice embryos were transformed with the 6 M-*PSY1* or Na-*PSY1* constructs together with the *PDS* gene driven by the barley endosperm-specific D-hordein promoter and the *Ubi1-hpt* selectable marker. The inclusion of PDS was necessary to boost precursors in the early steps of the carotenoid biosynthesis pathway so that we could evaluate PSY1 enzymatic activity by rapid visual analysis of the callus for the accumulation of orange pigmentation.

Callus lines representing several independent transformation events were analyzed by PCR using primers overlapping the promoter region and the *PSY1* and *PDS* transgenes (Supplementary Fig. 3). Most of the 24 lines transformed with the corrected promoter construct (Co-lines) yielded a PCR product corresponding to the 6 M-*PSY1* transgene, with the exception of lines Co-5, Co-32 and Co-66 where faint bands were observed, they were due to errors during gel loading. Subsequent to this observation, the polymerase chain reaction was repeated to validate the generation of the anticipated PCR product (Supplementary Fig. 3A-1). Furthermore, lines Co-5, Co-22, Co-23, Co-44, Co-60 and Co-66 lacked a PCR product corresponding to the *PDS* transgene (Supplementary Fig. 3A-2). Similarly, most of the 20 lines transformed with the Na-*PSY1* construct (Na-lines) yielded a corresponding PCR product, with the exception of lines Na-6, Na-10, Na-12, Na-13, Na-20 and Na-22 (Supplementary Fig. 3B-1). Lines Na-5, Na-6, Na-10, Na-12, Na-13, Na-16, Na-20 and Na-22 lacked a PCR product corresponding to the *PDS* transgene (Supplementary Fig. 3B-2). The presence of both transgenes (*PSY1* and *PDS*) was confirmed in 17 of the 24 lines transformed with the 6 M-*PSY1* construct and in 11 of those transformed with the Na-*PSY1* construct. We selected 14 lines carrying the 6 M-*PSY1* construct (lines Co-4, Co-19, Co-26, Co-28, Co-30, Co-34, Co-35, Co-37, Co-40, Co-47, Co-48, Co-79, Co-80 and Co-83) and seven carrying the Na-*PSY1* construct (Na-2, Na-3, Na-8, Na-9, Na-15, Na-25 and Na-26) for further analysis. The higher molecular weight band observed in the *PSY1* PCR lanes is due to the amplification of the endogenous *PSY1* gene with its intron. Lines carrying only one of the two transgenes were selected as negative controls: Co-23 (*PSY1* only), Co-32 (*PDS* only), Na-5 and Na-16 (*PSY1* only).

### Analysis of *PSY1* and *PDS* expression in callus transformed with the native and corrected *PSY1* promoter constructs

**Fig. 6:**
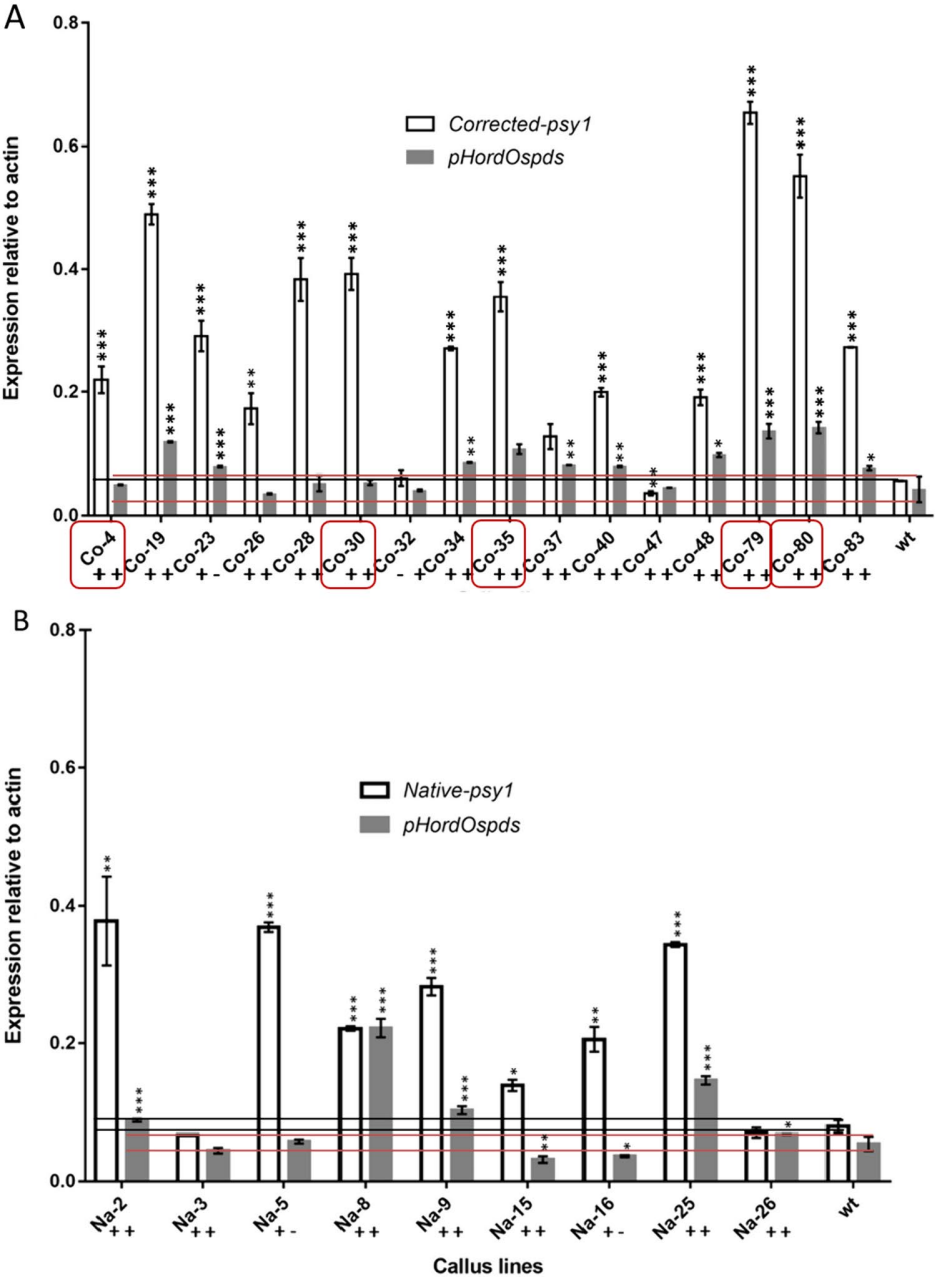
Accumulation of *PSY1* and *PDS* mRNA in independent transgenic callus lines. **A** Accumulation of *PSY1* and *PDS* mRNA in lines co-transformed with 6 M-*PSY1* and pHord-*PDS.* Line names begin with Co to indicate the corrected *PSY1* promoter. **B** Accumulation of *PSY1* and *PDS* mRNA in lines co-transformed with Na-*PSY1* and pHord-*PDS*. Line names begin with Na to indicate the native *PSY1* promoter. Expression levels were normalized to rice *actin*, as determined by qRT-PCR. Each value represents the mean of three experiments with standard errors. The + /− symbols below the bars indicate the presence or absence of the corresponding transgene (*PSY1* or *PDS*). The horizontal black and red lines indicate the variance in the expression level of the endogenous *PSY1* and *PDS* genes in wild-type callus, respectively. Statistical significance was determined using a one-way ANOVA (*p < 0.05, **p < 0.01, ***p < 0.001). Red boxes indicate lines that develop yellow/orange coloring (Fig. 8)

*PSY1* and *PDS* expression was analyzed by qRT-PCR (Fig. 6). The 6 M-*PSY1* lines produced on average twice as much *PSY1* mRNA as the Na-*PSY1* lines (a statistically significant difference, *p *< 0.05) whereas there was no significant difference in *PDS* mRNA levels between the two groups of lines (Fig. 6A, B). The levels of *PDS* mRNA were significantly (*p *< 0.05) higher than wild-type levels in lines Na-2, Na-8, Na-9, Na-25 and Na-26 (~ 44%), but significantly lower (*p* < 0.05) in lines Na-15 and Na-16 (~ 22%), and not significantly different in lines Na-3 and Na-5 (~ 22%), suggesting some natural variation in transgene expression between lines despite the use of the same promoter, again probably reflecting genomic position effects.

### Carotenoid content and composition in callus transformed with the native and corrected *PSY1* constructs

**Fig. 7:**
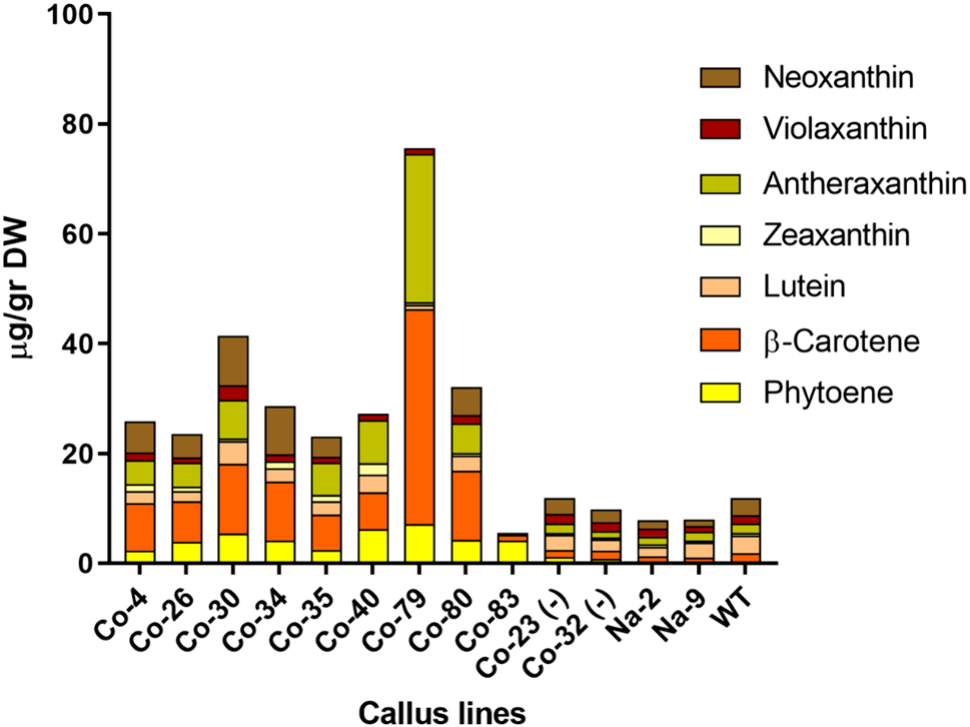
Carotenoid content and composition in transgenic rice callus lines. The carotenoid content and composition was determined in the corrected 6 M-*PSY1* lines (names beginning with Co), the corrected *PSY1* negative controls [Co-(-)] and the native (Na-*PSY1*) callus lines by HPLC. Values are means of three technical replicates ± standard errors. Final concentrations are presented as µg g^−1^ dry weight (DW)

**Fig. 8:**
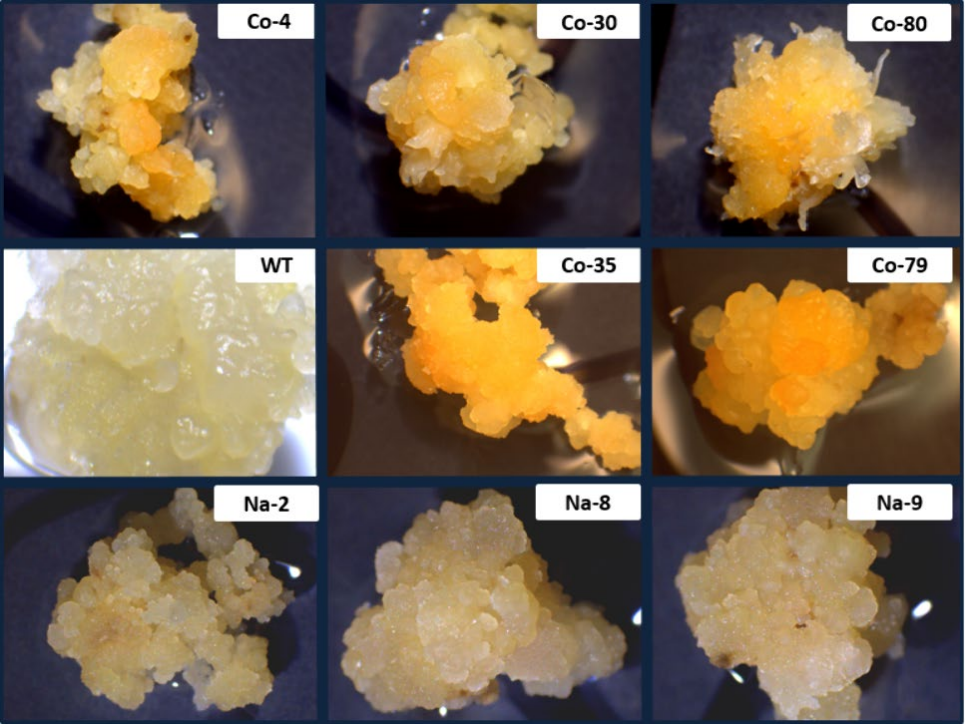
Color phenotype in 6 M-*PSY1* transgenic callus lines. Color phenotype of independent callus lines co-transformed with the 6 M-*PSY1* and pHord-*PDS* constructs, Na-*PSY1* and wild-type (WT) callus. The lines Co-4, Co-30, Co-80, Co-35 and Co-79 were selected on the basis of the color phenotype due to the reactivation of the carotenoid biosynthesis pathway and the accumulation of carotenoids

Embryo-derived rice callus is normally white because it accumulates negligible levels of carotenoids. However, many of the transformed callus lines developed a yellow or deep orange color. We analyzed the carotenoid content and composition of representative 6 M-*PSY1* lines as well as Na-*PSY1* lines lacking coloration (Fig. 7). All 6 M-*PSY1* lines showed an increase in phytoene levels compared to wild-type callus, with particularly high levels in lines Co-30 (5.38 µg g^−1^ dw), Co-40 (6.22 µg g^−1^ dw), Co-79 (7.13 µg g^−1^ dw), Co-80 (4.23 µg g^−1^ dw) and Co-83 (4.17 µg g^−1^ dw). The 6 M-*PSY1* lines with the most striking increase in total carotenoid levels acquired a characteristic orange color which was not homogeneously distributed (Fig. 8). This may reflect the developmental regulation of carotenoid metabolism in callus tissue where not all cells are at the same stage. A similar non-homogeneous colour phenotype has been reported in other studies of rice callus modified to accumulate carotenoids (Endo et al. 2019; Kim et al. 2013; Tzuri et al. 2015). Most lines (Co-83 was an exception) showed increases in the amount of β-carotene, zeaxanthin, antheraxanthin and neoxanthin. The levels of β-carotene were especially high in lines Co-30 (12.7 µg g^−1^ dw), Co-79 (39.17 µg g^−1^ dw) and Co-80 (12.62 µg g^−1^ dw). However, lutein and violaxanthin levels remained similar to wild-type levels. Most of these lines accumulated the highest levels of *PSY1* mRNA (Fig. 6) and the highest levels of β-carotene (Fig. 7). Two of the native-*PSY1* lines (Na-2 and Na-9) were tested together with two negative control *PSY1* lines (Co-23 and Co-32) but did not show any significance differences in carotenoid levels compared to wild-type callus.

## Discussion

SLEEPERs are defined herein as *silent latent endosperm-enabled promoters*, meaning that the configuration of *cis*-acting elements in the promoter is not permissive for gene expression in the endosperm. The rice *PSY1* gene was selected as a case study because it represents the first committed step in the carotenoid biosynthesis pathway and its activation in the endosperm leads to an easily discernable color phenotype, as long as downstream genes essential for carotenoid biosynthesis are also expressed. Activating the *PSY1* gene is not sufficient alone to fully enable carotenoid biosynthesis in the callus or endosperm, so we also included a phytoene desaturase to convert phytoene into ζ-carotene, based on our previous work showing that the expression of *PSY1* and *PDS* driven by endosperm-specific promoters is sufficient to enable the accumulation of carotenoids in rice callus and seeds (Bai et al. 2014, 2016; Zhu et al. 2008). Importantly, endosperm promoters are also active in embryogenic rice callus (Bai et al. 2014). This means that modified phenotypes generated by awakening SLEEPERs can be identified at the callus stage, before committing the time and labor required to regenerate plants. Proof-of-concept experiments to evaluate carotenoid expression in callus before producing transgenic plants have also been reported in sweet potato (*Ipomoea batatas*) for the overexpression of the *IbOr* gene (Kim et al. 2013) and in *Arabidopsis thaliana* for the functional analysis of *CmOr* haplotypes (Tzuri et al. 2015).

The *cis*-acting regulatory elements and corresponding transcription factors that control the expression of carotenogenic genes in rice endosperm are not fully understood. We therefore analyzed the rice *PSY1* promoter in silico looking for motifs in the first 2.5 kb upstream of the transcriptional start site resembling *cis-*acting elements commonly found in the promoters of other endosperm-specific genes. We identified six candidates, namely three P-boxes, one AACA motif, one GCN4-like motif and an O2 box, differing from the authentic sequences by 1–4 nucleotides. The P-box was the first motif shown to direct endosperm-specific expression in cereals and is found in the promoters of many genes encoding seed storage proteins (Kreis et al. 1985; Kawakatsu et al. 2008; Qu et al. 2008). The “endosperm box”, is a well-conserved sequence (Hartings et al. 1990) that consists of two independent elements: the P-box (TGDAAAG) and GCN4 motif (TGASTCA) (Hammond‐Kosack et al. 1993). The GCN4 motif also occurs separately, or in the presence of an AACA motif (Wu et al. 1998; Wu et al. 2000; Kawakatsu et al. 2008; Jin et al. 2019). Interestingly, mutations in the P-box were previously shown to reduce the expression of genes in the endosperm by ~ tenfold, whereas mutations in the GCN4 core motif reduce expression levels by fourfold, and those in the AACA motif cause a complete loss of promoter activity (Wu et al. 2000). The O2 box is 10 nucleotides in length with the sequence TCCACGTAGA (Izawa et al. 1993). The three main transcription factor families involved in endosperm-specific gene expression in cereals are DOF zinc fingers such as PBF, which binds the P-box, MYB family proteins that bind the AACA motif, and bZIP proteins that bind the GCN4 motif and the ACGT core sequence of the O2 box (Izawa et al. 1993; Juhász et al. 2011; Hernandez-Garcia and Finer 2014; Ding et al. 2021).

The increasing number and availability of plant genome sequences and bioinformatics tools has provided the datasets needed to characterize and understand the function of regulatory elements in the promoters of plant genes (Lescot et al. 2002; Bailey et al. 2009; Schnable et al. 2009; Schmutz et al. 2010; Brenchley et al. 2012). Transcriptional competence is influenced by the specificity, spacing and copy number of *cis*-acting regulatory elements (Ali and Kim 2019). It was, therefore, unclear whether the *PSY1* gene could be switched on in the embryogenic callus simply by “correcting” the near-miss elements or whether their juxtaposition, orientation, spacing and copy number would need to be adjusted too. Accordingly, we elected to test the principle by transforming rice embryogenic callus with different constructs driven by the native (uncorrected) promoter and two corrected versions with four (4 M) or all six (6 M) of the candidate SLEEPER motifs converted into their functional counterparts. The 4 M promoter (featuring three P-boxes and one AACA motif) resembled the promoter of the maize *BCH2* gene, which we previously upregulated by expressing the PBF and MYB transcription factors (Jin et al. 2019). We therefore hypothesized that a corrected *PSY1* promoter with three P-boxes and an AACA motif would probably be sufficient to drive reporter gene expression in rice. As anticipated, *GPF* was strongly expressed in callus and transgenic plants, confirming that the four motifs in the 4 M promoter are sufficient, their juxtaposition is satisfactory, and that the corresponding transcription factors are present in both native rice tissues.

Despite the success with *GFP*, the *PSY1* gene attached to the same 4 M promoter was not expressed. This result indicated there must be an intrinsic property that distinguishes the GFP and PSY1 systems downstream of the expression cassette, perhaps reflecting mRNA and/or protein stability. Indeed, GFP has been widely used for the analysis of *cis*-regulatory elements in the context of gene regulation precisely because it is a stable and reliable protein (Zhang et al. 2019). On the other hand, this outcome was not altogether surprising because the transcription factors that bind the P-box and AACA motif in the maize *BCH2* promoter did not induce native *PSY1* when overexpressed in maize, indicating that the two elements are not sufficient to drive adequate levels of *PSY1* expression (Jin et al. 2019). Like *BCH2*, the 5′-flanking region of Zm*PSY1* contains one AACA motif (−827 to −821), two P-boxes (−1221 to −1215 and −1341 to −1335) and one reverse and complementary P-box (−1600 to −1594). The conservation of such elements suggests they are functional, so they may be necessary but not sufficient for expression. We therefore also corrected the GCN4 motif and O2 box because these are bound by a different transcription factor than the P-box and AACA motif, so we theorized that these additional elements may be necessary to enhance the basal expression of *PSY1* enabled by the others. Accordingly, we found that the 6 M promoter was indeed transcriptionally competent, driving high levels of *PSY1* transgene expression in rice callus.

Similar results have been reported for other genetic constructs. In maize, six *cis*-acting elements (RY repeats, GCN4, P-box, Skn-1, ACGT and AACA) were fused to the *ZmBD1* promoter to generate a putative bidirectional promoter (Liu et al. 2018), resulting in the endosperm-specific expression of metabolic transgenes and the accumulation of anthocyanins in the endosperm. In soybean, a synthetic promoter was developed by adding repetitive G-box motifs to the relatively weak soybean glycinin promoter (*GmScream3*), leading to a significant increase in *GFP* expression (Zhang et al. 2019). Our 4 M promoter achieved similar results to these synthetic and hybrid promoters but the key difference is that our promoter was constructed by modifying the sequence of near-miss elements in their natural context rather than assembling a promoter from pre-formed active elements, which means that our results could in theory be replicated by genome editing. Notably, one of the transgenic rice lines transformed with the Na-*PSY1* construct produced low but detectable levels of GFP in some callus lines, probably reflecting the insertion of the cassette near a strong enhancer that might work in the same manner as an enhancer trap (Springer 2000).

The analysis of *PSY1* and *PDS* gene expression in lines transformed with the 6 M-*PSY1* constructs revealed higher levels of *PSY1* mRNA in lines transformed with the corrected *PSY1* construct and the accumulation of more carotenoids (mainly β-carotene) in the callus, resulting in an orange phenotype. However, there was no clear correlation between the appearance of the orange phenotype in these lines and the highly variable expression of *PDS*. The most likely explanation is that the variability in gene expression and carotenoid accumulation is due to a threshold effect, below which moderately increased carotenoid pathway activity is compensated by carotenoid degradation, and above which carotenoid levels exceed the rate of degradation and thus increase the steady-state levels of β-carotene and upstream intermediates. This phenomenon has been described in a non-green Arabidopsis callus system established to study carotenoid biosynthesis, degradation, and accumulation (Schaub et al. 2018). The authors found that carotenoid degradation was mostly non-enzymatic and selective for specific carotenoid types. They also identified factors controlling carotenoid catabolism and found no changes in the mutant callus, confirming the suitability of the Arabidopsis callus system for the analysis of carotenoid metabolism. The threshold of *PDS* expression required for downstream carotenoid synthesis, therefore, appears to be so low that *PSY1* is the main bottleneck in almost all transgenic lines. Even so, the accumulation of carotenoids confirmed our hypothesis that small modifications in the promoter of the *PSY1* gene were sufficient to restore gene expression and PSY1 enzyme activity in the callus, validating the SLEEPER approach.

The concept of in situ promoter modification by genome editing in plants was first demonstrated by the creation of an allelic series in the *CLAVATA3* gene controlling fruit size in tomato (Rodriguez-Leal et al. 2017). Subsequently, the same principle has been used to modulate the activity of natural and synthetic soybean promoters (Zhang et al. 2019), the rice *Waxy* promoter as a means to control grain quality (Huang et al. 2020; Zeng et al. 2020), and the rice *Hd2* promoter to delay flowering (Liu et al. 2021). However, these studies set out to modulate genes that were already expressed, so the *cis*-acting elements were already organized in a manner that was permissive for gene expression. We are not aware of any previous report in which the same approach has been used to activate a silent endogenous promoter (SLEEPER) by correcting short motifs that just happen to resemble genuine *cis*-acting elements or may be evolutionary remnants of a regulatory cassette that has drifted over time following gene duplication events. This justifies our cautious approach in which we first tested the corrected promoters as conventional transgenes using standard transformation methods.

We conclude that the correction of near-miss sequences to make them match the genuine *cis*-acting elements found in rice endosperm-specific promoters was sufficient to switch on *GFP* gene expression in embryogenic callus and the endosperm of transgenic plants, as well as *PSY1* expression in callus. This confirms our hypothesis that the SLEEPER strategy is suitable for the ectopic activation of silent metabolic genes. Our results offer further insights into the bottlenecks preventing carotenoid biosynthesis in rice endosperm and the potential of in situ promoter modification for the ectopic activation of metabolic pathways for purposes such as nutritional enhancement in grain, the improvement of agronomic traits, or the development of rice callus or seeds as a production platform for valuable natural products.

## Experimental procedures

### Bioinformatic analysis of the native and corrected *PSY1* promoters

We screened the 2.5-kb upstream promoter region of the rice (*Oryza sativa* cv. Nipponbare) *PSY1* gene (GenBank AP014962.1, Supplementary Fig. 1) for close matches to genuine endosperm-specific regulatory motifs but containing up to four mismatches rendering them inactive. Six candidate motifs were identified by using the functional elements as BLAST queries (Table 1). Our in silico promoter analysis was based on the variety Nipponbare but we switched to a local Spanish variety (Bomba) for the callus experiments due to supply issues during the COVID-19 pandemic, having established that the sequence of the *PSY1* gene and the upstream 120-bp promoter region were identical in both cultivars (OR590619). The native and corrected sequences were compared using PLACE software to predict the outcome of the modifications.

### Cloning and preparation of the expression constructs

The *GFP* gene was isolated from the 35S-*eGFP*-nosT construct (Yutaka Kodama; Addgene plasmid #80127; http://n2t.net/addgene:80127). The *PSY1* and *PDS* coding regions were cloned directly from rice leaf mRNA by RT-PCR using the forward and reverse primers listed in Supplementary Table 1. The primers were based on the coding sequences in GenBank (accession numbers FJ971175 and XM_015777614). The RT-PCR products were transferred to vector pGEM-T easy (Promega, Madison, WI, USA) to generate final vectors pGEM-*OsPSY1* and pGEM-*OsPDS*. The integrity of the final vectors was confirmed by sequencing (STABVIDA, Caparica, Portugal).

The native and corrected *PSY1* promoters were synthesized by Synbio Technologies (Monmouth Junction, NJ, USA). The sequences were released from the source vectors using restriction enzymes HindIII and XhoI, separated by 1% agarose gel electrophoresis and purified using the QIAquick PCR Purification Kit (Qiagen, Hilden, Germany). After purification, an NcoI restriction site was added by PCR at the 5′ end of the 4 M and native promoter sequences for joining to the 35S-*GFP*-TNos backbone without the CaMV 35S promoter (Addgene plasmid #80,127) by ligation using 5 U/µL T4 DNA ligase (Thermo Fisher Scientific, Waltham, MA, USA). The 4 M, 6 M and native sequences were also transferred to vector pUC57, which had been linearized with HindIII and XhoI. The pGEM-*OsPSY1* vector was digested with XhoI and EcoRI and the cDNA was transferred to the pUC57 vectors containing the promoter fragments, which had been digested with the same enzymes. The pGEM-*OsPDS* vector was digested with XbaI and SacI and the cassette was transferred to the pHorp-P vector  (Nicolaou and Sorensen 1996) containing the endosperm-specific barley D-hordein promoter and the rice ADP-glucose pyrophosphorylase terminator. A third vector was prepared containing the *Ubi1* promoter/first intron and the selectable marker *hpt* encoding hygromycin phosphotransferase. The integrity of all constructs was verified by sequencing as above.

### Transformation, recovery and growth of transgenic callus

Mature rice zygotic embryos (*O. sativa* cv. Bomba, provided by Illa de Riu, Tarragona, Spain) were transformed by direct DNA transfer followed by regeneration under hygromycin selection as previously described (Saba-Mayoral et al. 2022). Fast-growing embryogenic callus on the scutellum was transferred to fresh Murashige and Skoog selection medium (MSS) and subcultured every 2 weeks under the same conditions. After the second subculture, when the callus started to develop the characteristic orange phenotype, 200-mg samples were taken for RNA extraction and qRT-PCR. In subsequent subcultures, callus samples were taken from each line and frozen at −80 °C until 16 g was available for metabolomic analysis.

### Molecular characterization of transgenic callus lines

DNA was isolated from the transgenic callus lines as previously described (Creissen and Mullineaux 1995). Briefly, 300-mg samples of callus were ground in a porcelain mortar under liquid nitrogen and the powder was transferred to a 2-mL Eppendorf tube containing 750 µL extraction buffer (100 mM Tris–HCl pH 8, 500 mM NaCl, 50 mM EDTA) and 75 µL 20% (w/v) SDS. The mixture was vortexed for 10 min and incubated at 65 °C for 1 h before precipitation with one volume of 25:24:1 phenol–chloroform-isoamyl alcohol followed by centrifugation (13,000×*g*, 5 min, room temperature). The supernatant was mixed with 6 µL RNase (10 mg mL^−1^) and incubated for 30 min at 37 °C. After another round of phenol extraction, the supernatant was mixed with an equal volume of isopropanol to precipitate the DNA. After centrifugation (13,000×*g*, 10 min, 4 °C), the DNA pellet was washed with 1 mL 70% ethanol and dissolved in 100 µL Millipore water. The DNA was quantified using a Nanodrop 2000c spectrophotometer (Thermo Fisher Scientific). The DNA was diluted to 100 ng/µL and the *PSY1* and *PDS* genes were amplified by PCR in a 20-µL reaction containing 2 µL DreamTaq Green Buffer 10×with 20 mM MgCl_2_, 0.2 µL dNTPs (10 µM), 0.2 µL of the forward and reverse primers (10 µM), 0.125 µL DreamTaq DNA polymerase (5 U/µL), and 1 mg of DNA (all reagents from Thermo Fisher Scientific) topped up with Millipore water. The template for the *GFP* gene was denatured at 95 °C for 4 min followed by 30 cycles of denaturation at 95 °C for 45 s, annealing at 63 °C for 45 s, and extension at 72 °C for 45 s, and a final extension step at 72 °C for 3 min. The template for the *PSY1* gene was denatured at 95 °C for 4 min followed by 30 cycles of denaturation at 95 °C for 45 s, annealing at 58 °C for 45 s, and extension at 72 °C for 45 s, and a final extension step at 72 °C for 3 min. The template for the *PDS* gene was denatured at 95 °C for 4 min followed by 30 cycles of denaturation at 95 °C for 45 s, annealing at 59 °C for 45 s, and extension at 72 °C for 45 s, and a final extension step at 72 °C for 3 min. The *GFP* gene was amplified using forward primer 5′-GAT CGA TGC TGA GCT GCA ACC AAG-3′ (FW5), which anneals within the last 100 bp of the promoter, and reverse primer 5′-TTA CTT GTA CAG CTC GTC CAT GCC GT-3′ (RV5), which anneals at the end of the *GFP* gene (Supplementary Fig. 4). The *PSY1* promoter and gene sequences were amplified using forward primer 5′-GAT CGA TGC TGA GCT GCA ACC AAG-3′ (FW1), which anneals within the promoter, and reverse primer 5′-GCG TCC GGT GAA GAG ATC ATC AAG C-3′ (RV1), which anneals at the end of the *PSY1* gene (Supplementary Fig. 4). The *PDS* gene sequence was amplified using forward primer 5′-ACT AAC ACA GCC GTG TGC ACA TAG-3′ (FW3), which anneals within the pHord promoter, and reverse primer 5′-GAA TGC ACT GCA TGG ATA ACT CAT CGA-3′ (RV3), which anneals within the *PDS* gene (Supplementary Fig. 4). The integrity of the genes was confirmed by independent PCRs overlapping the first amplicons, with forward primers annealing within the first 100 bp of the gene and reverse primers annealing within the terminator (FW2/RV2 for *PSY1* and FW4/RV4 for *PDS*; Supplementary Fig. 4). PCR products were resolved by 1% agarose gel electrophoresis in 1×TAE buffer.

### Gene expression analysis

Total RNA was extracted from callus tissue using the phenol:chloroform:isoamyl alcohol (25:24:1) method, precipitated with 4 M LiCl (Creissen and Mullineaux 1995) and quantified using a Nanodrop 2000c spectrophotometer. We used 1 μg of total RNA as the template for first-strand cDNA synthesis with Ominiscript Reverse Transcriptase (Qiagen) and dsDNase (Thermo Fisher Scientific) in a 20-μL reaction. The cDNA was amplified by qPCR on a CFX96 system (Bio-Rad Laboratories, Hercules, CA, USA). Each 25-µL reaction contained 10 ng cDNA, 1× iQ SYBR Green Supermix (Bio-Rad) and 0.2 µM of each primer. To calculate relative expression levels, serial dilutions of each RNA sample (0.2–125 ng) were used to produce standard curves for each gene. Triplicate reactions were prepared in 96-well optical reaction plates with the following profile: denaturation for 3 min at 95 °C followed by 39 cycles of 95 °C for 10 s, 59  C for 30 s, and 72 °C for 20 s. Amplification specificity was confirmed by melt curve analysis of the final PCR products in the temperature range 50–90 °C with fluorescence acquired after each 0.5 °C increment. The fluorescence threshold value and gene expression data were calculated using CFX96 system software. The *GFP* gene was amplified using forward primer 5′-GAA GCA GCA CGA CTT CTT CAA-3′ and reverse primer 5′-TAT AGA CGT TGT GGC TGT TGT AGT-3′. The *PSY1* gene was amplified using forward primer 5′-TAT GCT CAT GAC GGA GGA CC-3′ and reverse primer 5′-GCA TCA AGC ATG TCG TAG GG-3′. The *PDS* gene was amplified using forward primer 5′-TTG CGG GAC AAC TTC CTA CT-3′ and reverse primer 5′-AAC AAC CTG TAG AGC ACC GA-3′. The rice *actin* gene (X15865.1) was amplified using forward primer 5′-TCA TGT CCC TCA CAA TTT CC-3′ and reverse primer 5′-GAC TCT GGT GAT GGT GTC AGC-3′.

### Protein extraction and western blot analysis for the detection of GFP

Total rice protein extracts were prepared by grinding 10–15 seeds in liquid nitrogen and thawing the powder in 0.2–0.4 mL of extraction buffer: 20 mM Tris–HCl pH 7.5, 5 mM ethylenediaminetetraacetic acid (EDTA), 0.1% Tween-20, 0.1% sodium dodecylsulfate (SDS), 2 mM phenylmethanesulfonylfluoride (PMSF). The mixture was vortexed for 1 h at 4 °C. Cell debris was removed by centrifugation (15,000×*g*, 20 min, 4 °C) and the supernatant was collected and stored at −80 °C. The protein concentration in the supernatants was determined using the Bradford method (AppliChem, Darmstadt, Germany). Total rice protein (80 mg) was fractionated by denaturing sodium dodecylsulfate polyacrylamide gel electrophoresis (SDS-PAGE) in gels containing 10% SDS at 200 V for 60 min, and then electro-transferred to an Immobilon FL polyvinylidene difluoride (PVDF) membrane (Merck, Darmstadt, Germany) using a semidry transfer apparatus (Bio-Rad) at 20 V for 45 min. The membrane was immersed in 5% non-fat milk in TBST (0.2 M Tris–HCl pH 7.6, 1.37 M NaCl, 0.1% Tween-20) for 1 h at room temperature. Membranes were incubated with anti-GFP polyclonal antibody SAB4301138 (Sigma-Aldrich, St Louis, MO, USA) diluted 1:2000 in 5% non-fat milk in TBST overnight at 4 °C, then rinsed three times for 10 min in TBST. The membranes were subsequently incubated with an alkaline phosphatase-conjugated goat anti-rabbit secondary antibody (Sigma-Aldrich) diluted 1:5000 in 2% non-fat milk in TBST for 1 h at room temperature followed by three 10-min rinses in TBST. Signals were detected using SIGMAFAST BCIP/NBT tablets (Sigma-Aldrich).

### Confocal microscopy to detect GFP activity

Seeds from rice lines expressing 4 M-*GFP* were analyzed by confocal microscopy to detect GFP and reveal its subcellular localization. Seeds from WT rice and transgenic lines expressing Na-*GFP* were used as negative controls. Fresh small seed pieces (1×10 mm) were fixed with 2% paraformaldehyde in 0.1 M sodium phosphate buffer (pH 7.2) before semi-thin Sects. (30–40 µm) were prepared using a CM3050 S Research Cryostat (Leica Microsystems, Wetzlar, Germany). The sections were collected on glass microscope slides coated with poly-l-lysine and 1024×1024 pixel images were captured using an FV1000 laser scanning confocal microscope (Olympus, Hamburg, Germany) fitted with a 100 × NA 1.40 UPlansApo (oil) objective. For GFP imaging, the excitation wavelength was 488 nm provided by a multiline argon laser. Three independent transgenic rice lines were analyzed for each event.

### Carotenoid analysis

We combined UPLC (Waters Acquity, Watford, UK) and HPLC–PDA (Waters Alliance, Watford, UK) systems to analyze freeze-dried callus material transformed with the Na-*PSY1* and 6 M-*PSY1* constructs, and the same amount of WT callus as a control. Carotenoids were extracted from 10 mg of powdered tissue in 500 μL 50/50 water/methanol and vortexed for 30 min at room temperature. After mixing with 500 μL chloroform and separating the phases by centrifugation (15,300×*g*, 3 min, 4 °C), the lower phase was collected, evaporated, and re-dissolved in HPLC-grade ethyl acetate for UPLC-PDA analysis on an ethylene-bridged hybrid (BEH C18) column (2.1×100 mm, 1.7 mm) and a BEH C18 VanGuard pre-column (2.1×50 mm, 1.7 mm). The samples were eluted in a gradient of mobile phase A (50/50 methanol/water) and mobile phase B (75/25 acetonitrile/ethyl acetate) at a flow rate of 0.5 mL/min. All solvents were passed through a 0.2-mm filter before use. The gradient started at 30% A for 0.5 min and was then stepped to 0.1% A for 5.5 min and to 30% A for the last 2 min. The column temperature was maintained at 30 °C and the sample temperature at 8 °C. Continuous online scanning was performed across the UV/visible range from 250 to 600 nm, using a Waters extended wavelength photodiode array detector (Nogueira et al. 2013). Carotenoid identity was confirmed by HPLC–PDA (Fraser et al. 2000). Carotenoids were quantified by comparison to dose–response curves prepared from authentic standards.

### Statistical analysis

All experimental data were analyzed using SPSS Statistics for Windows v28.0 (IBM, Armonk, NY, USA). Mean values for each measured parameter were compared using a one-way ANOVA.

### Accession numbers

FJ971175.1: *Oryza sativa* Japonica Nipponbare *PHYTOENE SYNTHASE *(PSY1) gene, complete cds. AP014962.1: Nipponbare chromosome 6. AK070716: *Oryza sativa PSY1* cDNA. OR590619: *Oryza sativa* cv. Bomba *PSY1*120-bp upstream promoter region. X15865.1: *Oryza sativa actin* (*RAc1*) gene sequence.

### Supplementary Information

Below is the link to the electronic supplementary material.

Supplementary file1 (DOCX 963 KB)

## Data Availability

All data generated or analysed during this study are included in this published article [and its supplementary information files].
