# Supplementary material for: Activation of the native PHYTOENE SYNTHASE 1 promoter by modifying near-miss cis-acting elements induces carotenoid biosynthesis in embryogenic rice callus
Source: Plant Cell Rep. 2024 Apr 17;43(5):118. doi: 10.1007/s00299-024-03199-7 (PMC11024007; doi:10.1007/s00299-024-03199-7)
Supplement: Supplementary file 1 — Supplementary file1 (DOCX 963 KB) [file 299_2024_3199_MOESM1_ESM.docx]

**Supplementary Figures and Tables**

**
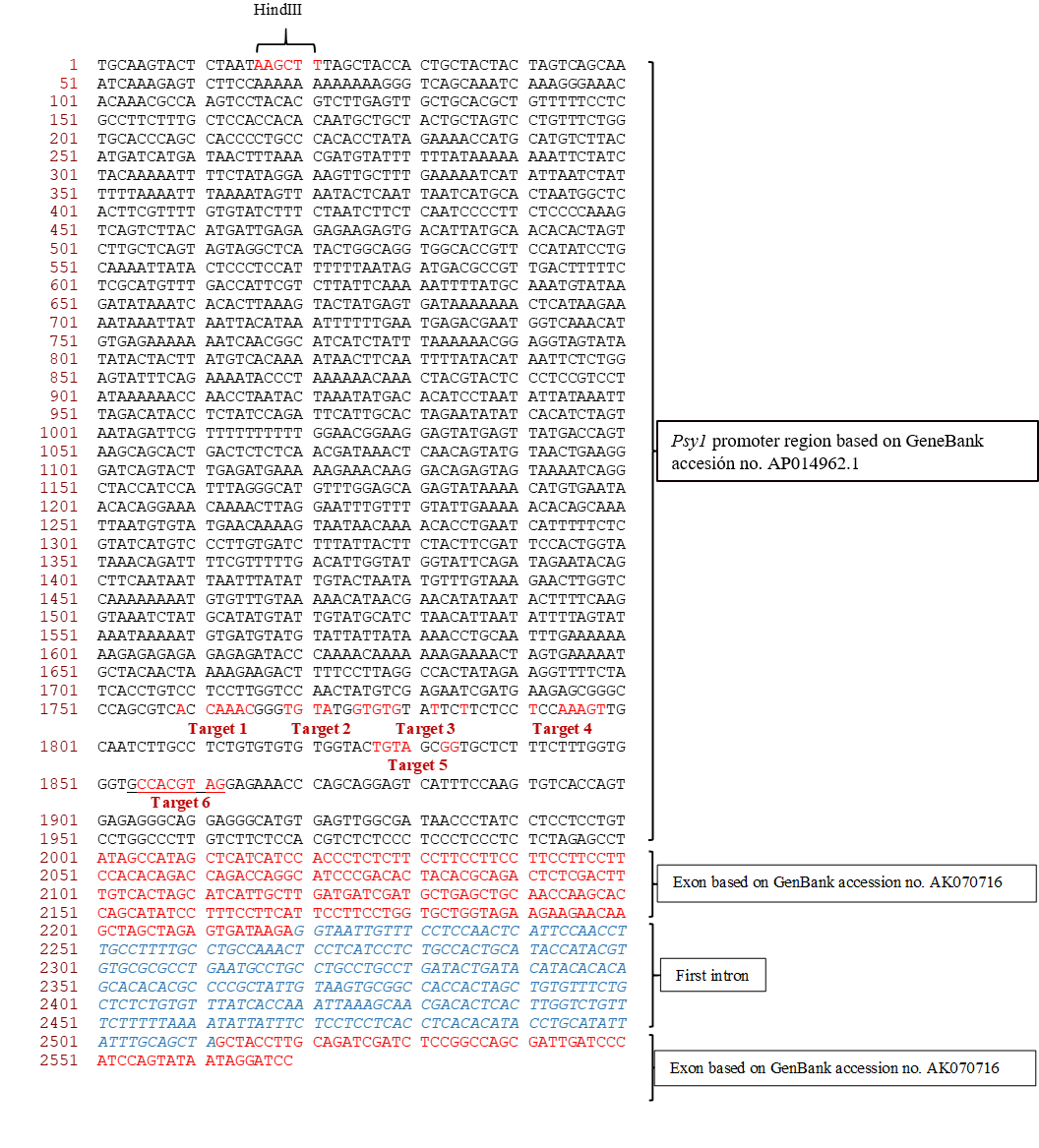
**

**Supplementary Figure 1. The sequence of the *PSY1* promoter and part of the gene.**  The sequence of the rice (*O. sativa* cv Nipponbare) *PHYTOENE SYNTHASE 1* (*PSY1*) promoter (GenBank AP014962.1) and part of the cDNA (GenBank AK070716), with exons in red and the first intron (positions 2219–2511) in blue italics. The six target motifs in the promoter are shown in red and underlined.


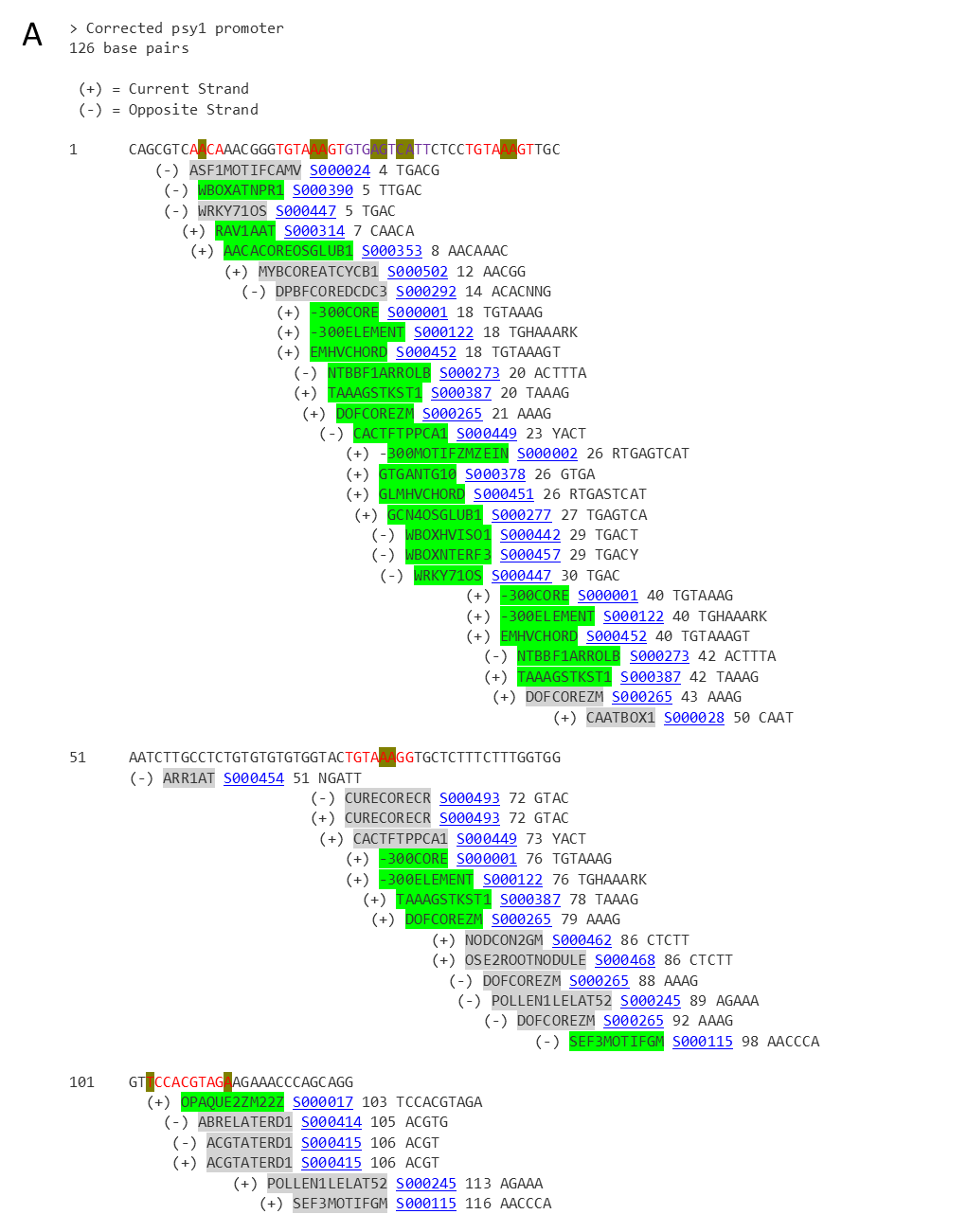


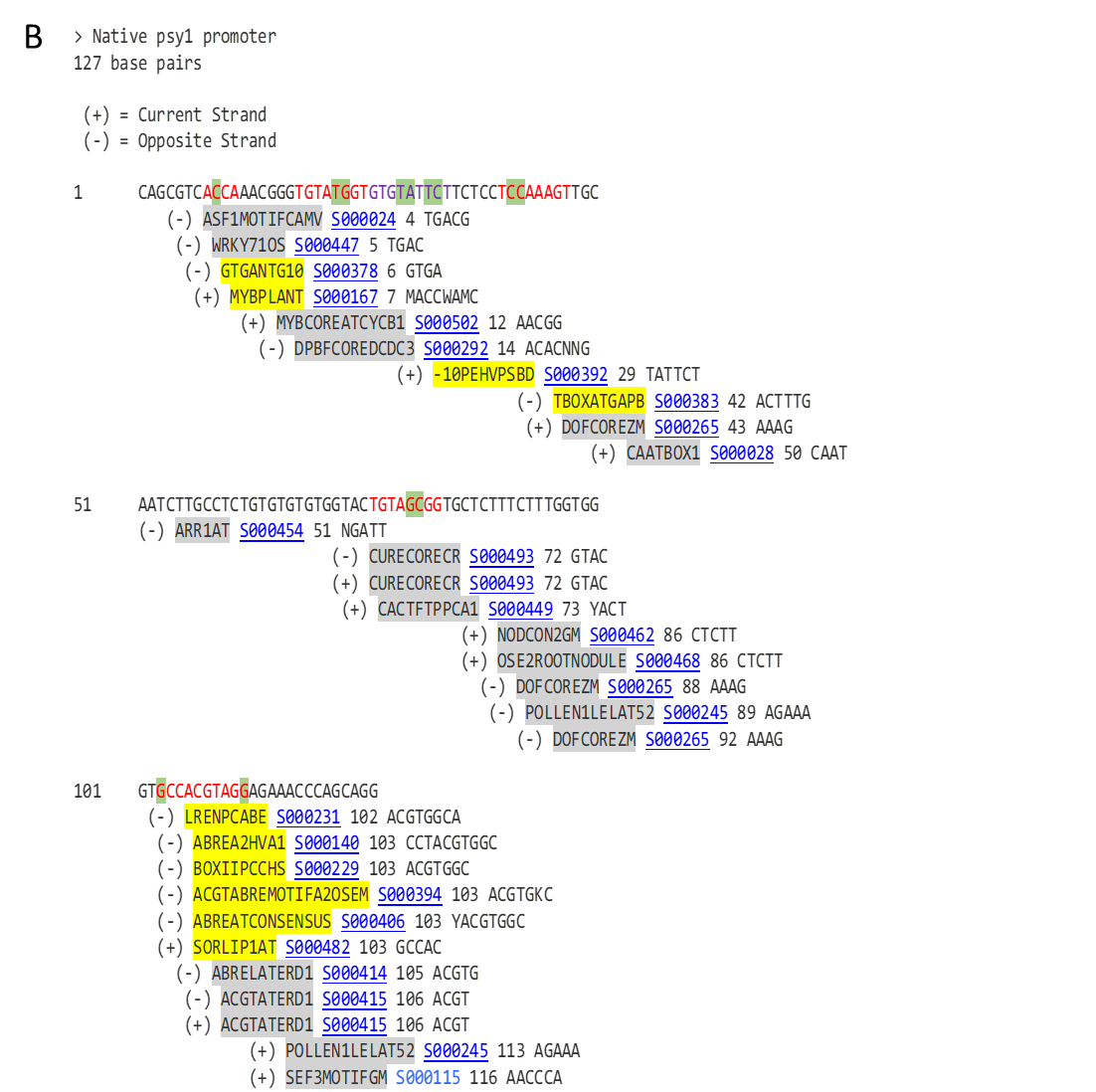


**Supplementary Figure 2. Analysis of the –300 region of the rice *PSY1* promoter (~120 bp) *in silico* to identify potential *cis-*acting regulatory elements.** Analysis of (A) the native and (B) 6M corrected sequences of ~120 bp of the *PSY1* promoter (–300 region) using PLACE (https://www.dna.affrc.go.jp/PLACE). Letters in red and purple indicate the motifs to be corrected. The dark green highlight indicates the specific bases to be corrected in the native sequence (A), and those that have already been changed in the corrected sequence (B). The bright green highlight indicates the presence of motifs exclusively in the corrected sequence. The yellow highlight indicates the presence of motifs exclusively in the native sequence. The gray highlight indicates motifs common to both sequences.

**
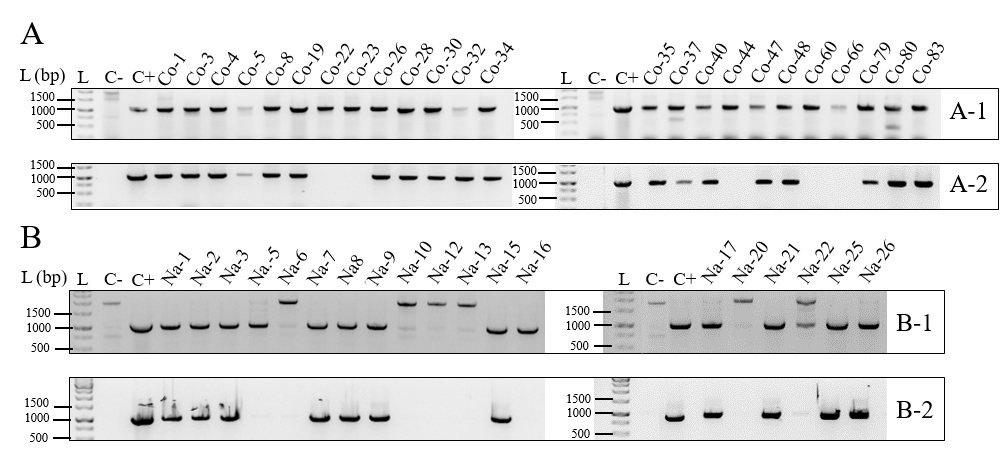
**

**Supplementary Figure 3. Analysis of genomic DNA from callus lines transformed with the native and corrected *PSY1* and pHord-*PDS* constructs.** (A) PCR amplification of genomic DNA from independent callus lines containing (A1) the 6M-*PSY1* and (A2) pHord-*PDS* constructs. Line names begin with Co to indicate the corrected *PSY1* promoter. (B) PCR amplification of genomic DNA from callus lines containing (B1) the Na-*PSY1* and (B2) pHord-*PDS* constructs. Line names begin with Na to indicate the native *PSY1* promoter. L = 1-kb DNA Ladder. H_2_O = negative control. C^–^ = wild-type rice plant (negative control). Lane C^+^ = plasmid DNA (positive control). The anticipated sizes of the *PSY1* and *PDS* amplicons are 1100 and 1041 bp, respectively. The larger band observed in the *PSY1* PCR lanes is due to the amplification of the endogenous *PSY1* gene with its intron.


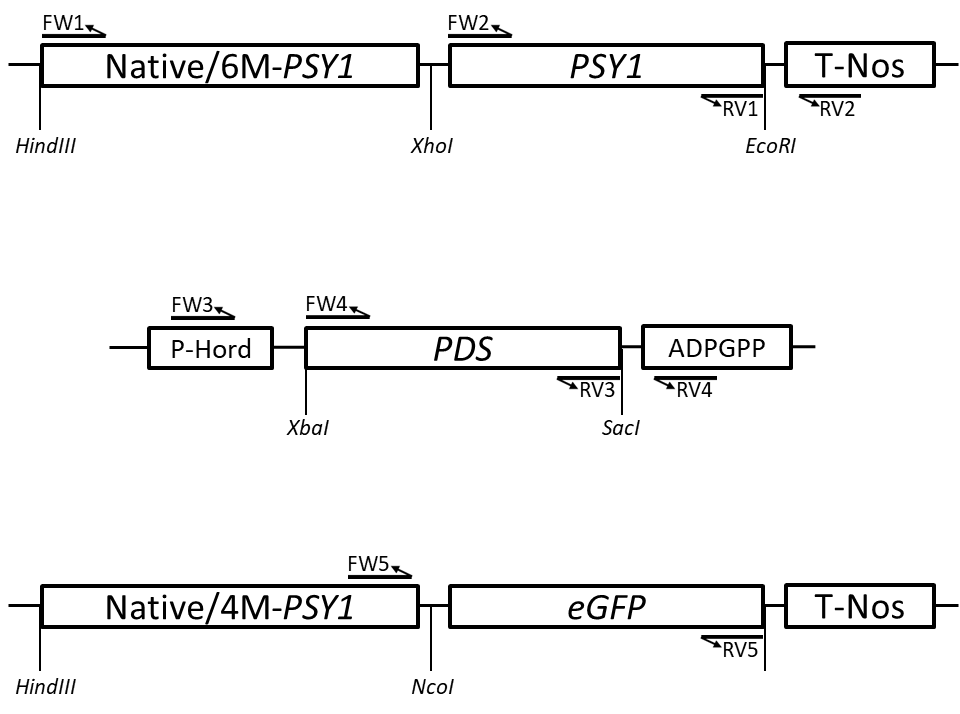


**Supplementary Figure 4. Schematic representation of the different primers and restriction sites used to clone the *GFP,* *PSY1* and *PDS* transgenes.** Vertical black lines indicate restriction sites and arrows indicate primer annealing sites.

| **Gene** | **Forward sequence (5′→3′)** | **Reverse sequence (5′→3′)** |
| --- | --- | --- |
| ***PSY1*** | CTCGAGATGGCGGCCATCACGCTCCTA | GAATTCCTACTTCTGGCTATTTCTCAGTGA |
| ***PDS*** | TCTAGAATGGATACTGGCTGCCTGTCATC | GAGCTCCTAGGAGGCAACAGGAACTTCA |

**Supplementary Table 1 Primers used to amplify and clone the rice *PSY1* and *PDS* genes**. Restriction sites for XhoI (5′-CTCGAG-3′), EcoRI (5′-GAATTC-3′), XbaI (5′-TCTAGA-3′) and SacI (5′-GAGCTC-3′) are underlined.
